# Inflammation and Myeloid Cells in Cancer Progression and Metastasis

**DOI:** 10.3389/fcell.2021.759691

**Published:** 2022-01-21

**Authors:** Jenying Deng, Jason B. Fleming

**Affiliations:** ^1^ Department of Surgical Oncology, The University of Texas MD Anderson Cancer Center, Houston, TX, United States; ^2^ H. Lee Moffitt Cancer Center, Department of Gastrointestinal Oncology, Tampa, FL, United States

**Keywords:** inflammation, cancer, myeloid cells, neutrophils, MDSCs, macrophages

## Abstract

To date, the most immunotherapy drugs act upon T cell surface proteins to promote tumoricidal T cell activity. However, this approach has to date been unsuccessful in certain solid tumor types including pancreatic, prostate cancer and glioblastoma. Myeloid-related innate immunity can promote tumor progression through direct and indirect effects on T cell activity; improved understanding of this field may provide another therapeutic avenue for patients with these tumors. Myeloid cells can differentiate into both pro-inflammatory and anti-inflammatory mature form depending upon the microenvironment. Most cancer type exhibit oncogenic activating point mutations (ex. P53 and KRAS) that trigger cytokines production. In addition, tumor environment (ex. Collagen, Hypoxia, and adenosine) also regulated inflammatory signaling cascade. Both the intrinsic and extrinsic factor driving the tumor immune microenvironment and regulating the differentiation and function of myeloid cells, T cells activity and tumor progression. In this review, we will discuss the relationship between cancer cells and myeloid cells-mediated tumor immune microenvironment to promote cancer progression and immunotherapeutic resistance. Furthermore, we will describe how cytokines and chemokines produced by cancer cells influence myeloid cells within immunosuppressive environment. Finally, we will comment on the development of immunotherapeutic strategies with respect to myeloid-related innate immunity.

## Introduction

With solid tumors, the TME is complex and contains cancer, immune and stromal cells within a confined, three-dimensional space. In many solid tumors, the stromal compartment compromises up to 60–80% of the tumor mass which produces an microenvironment rich in ECM and inflammatory cells (Henke et al., 2019). Laboratory investigation and clinical studies have demonstrated that the immune system, including innate and adaptive-related immune cells in the tumor microenvironment, plays a powerful role in cancer progression. Data from the rapidly advancing field of cancer immunology has shown that cancer cells recruit host myeloid cells into the tumor microenvironment where they can create an immune suppressive or stimulatory environment that positively or negatively influences cancer progression and metastasis (Gregory and McGarry Houghton, 2011; Noy and Pollard, 2014; Alizadeh et al., 2015; Papageorgis and Stylianopoulos, 2015; Patel et al., 2018). Improved understanding of the role of this lineage of immune cells is critical for the continued development of successful immunotherapeutic strategies against cancer. In this review, we will focus on innate-myeloid cells in the TME and discuss current understanding of mechanisms by which these cells promote cancer progression.

## The Role of Myeloid Cells in Cancer Pathogenesis

**FIGURE 1:**
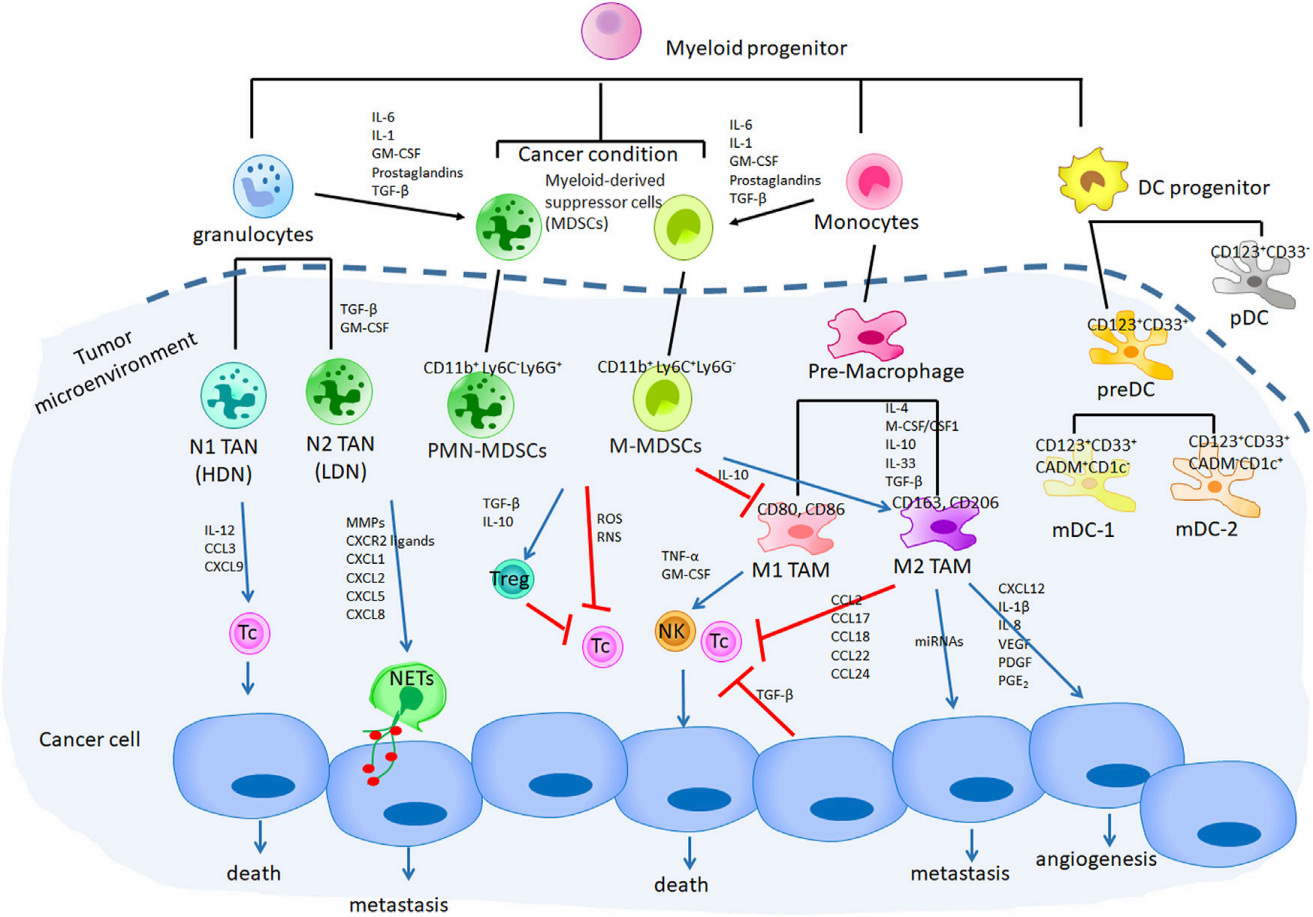
The role of myeloid cells in cancer progression through directly with cancer cells and indirectly regulated cancer immune evasion that promotes tumor growth. Cancer cells produce secretome to induced myeloid progenitor cells to form MDSCs and accumulated within the TME, which regulated TANs and TAMs switch to an anti-inflammatory status (N2 TAN and M2 TAMs). N2 TAN (PMN-MDSCs) directly induced NETs through CXCR2 pathway to promote cancer metastasis. M2 TAMs-related CXCL12, IL-1β, IL-8, VEGF, PDGF, PGE2 as well as miRNA regulated cancer angiogenesis and metastasis. In addition, both MDSCs and M2 TAMs induced Tregs generation and inhibited cytotoxic T and NK cells activity through their-related soluble factors.

Myeloid cells, including granulocytes, monocytes, macrophages, myeloid dendritic cells (mDCs), play an important role in recognition of pathogen and driving the innate immune response (Arandjelovic and Ravichandran, 2015; Lavin et al., 2015; Merad et al., 2013). The role of myeloid cells in cancer progression occurs through direct and indirect interaction with cancer cells (Figure 1) (Qian and Pollard, 2010; Gabrilovich et al., 2012; Broz and Krummel, 2015). The numerous subtypes of myeloid cells have each been demonstrated to regulate tumor pathogenesis; important subtypes include monocytes-derived macrophages, dendritic cells (DCs), granulocytes-derived neutrophils and Myeloid-derived suppressor cells (MDSCs).

### Macrophages

Macrophages are formed by the differentiation of monocytes after tissue injury, infection and inflammation in order to remove invading pathogens, apoptotic cells and debris, thus maintaining tissue integrity. Depend on its phenotypes and functions, the macrophage has been divided into 2 subgroups: M1 (the classically activated macrophages which exhibit proinflammatory effect) and M2 (the alternatively activated macrophages which exhibit anti-inflammatory effect) (Mackaness, 1962; Liu et al., 2014). M1 macrophages express high levels of MHC class II and costimulatory molecules, including CD80, and CD86; in contrast, M2 macrophages contain upregulated levels of human scavenger receptors, including CD163 and CD206 (Duan and Luo, 2021). In cancer, tumor-associate macrophages (TAMs) change status (M1 or M2) depending upon TME factors (Murray et al., 2014). M1 TAMs are initially activated by TNF-α or granulocyte–macrophage colony-stimulating factor (GM-CSF) followed by activation of Toll-like receptor signaling pathways; M1 TAMS can then recruit the cytotoxic CD8^+^ T and NK cells to kill the tumor cells (Mosser and Edwards, 2008). During tumor progression TAMs often switch to an anti-inflammatory M2 status after exposure to cytokines within the TME including: IL-4, M-CSF/CSF1, IL-10, IL-33, IL-21, and TGF-β. Importantly, TGF-β also inhibits cytotoxic T cell and NK cells-mediated anti-tumor immune response (Lawrence and Natoli, 2011; Noy and Pollard, 2014; Ruffell and Coussens, 2015). Translational studies suggest that the ratio of M1/M2 of TAMs within the primary tumor is associated with tumor stage and patient overall survival. For example, a lower M1/M2 ratio of TAMs within examined tumors is statistically associated with more advanced tumor stage and poor overall survival in ovarian cancer (Zhang et al., 2014; Yuan et al., 2017), pancreatic cancer (Di Caro et al., 2016; Yu et al., 2019), lung cancer (Wu et al., 2016), and gastric cancer (Wang et al., 2016).

TAMs are involved directly and indirectly in cancer progression through promotion of angiogenesis, cancer cell invasiveness and metastasis (Duan and Luo, 2021). Studies suggest that M2 TAMs, but not M1 TAMs, enhance tumor hypoxia that promotes angiogenesis by driving transcription of angiogenesis-associated genes, such as VEGF, PDGF, and PGE_2_ (Squadrito and De Palma, 2011; Jetten et al., 2014); importantly, failure of anti-angiogenic treatments by VEGF inhibitors is caused by the induction of other compensatory pro-angiogenic factors secreted by Tie2-expressing CD11b^+^ monocytes infiltrating tumor tissues (Belgiovine et al., 2016). Other TAM-mediated proangiogenic molecules, including CXCL12, IL-1β, IL-8, and Sema4d, recruit or activate endothelial cells (Murdoch et al., 2008; Qian and Pollard, 2010) that respond to growth factor signaling to form new blood vessels within the TME (Gurevich et al., 2018; Ramirez-Pedraza and Fernández, 2019).

TAMs also promote cancer invasiveness and metastasis by expressing proteinase, cathepsin, urokinase, and matrix remodeling enzymes that break down the tumor extracellular matrix (ECM) (Wynn et al., 2013). TAMs not only degrade the ECM but also promote epithelial–mesenchymal transition and invasiveness of tumor cells through secretion of TGF-β and growth factors, such as EGF analogs (Kessenbrock et al., 2010; Mason and Joyce, 2011; Bonde et al., 2012; Hanahan and Coussens, 2012). Recent studies have demonstrated that exosomes, which contain miRNAs released from M2 macrophages, can increase levels of TGF-β and upset the balance of Treg/Th17 cells within the TME to drive metastasis in various cancer types (Zhou et al., 2018; Lan et al., 2019; Yin et al., 2019).

Both pro- and anti-inflammatory cytokine secretion which influence cancer progression have been identified as originating from different types of TAMs within the tumor microenvironment. CXCL10, CXCL11, and CCL5 secreted by M1 TAMs, which recruit Th1, Th17, and cytotoxic T cells, exhibit anti-tumor effect. In contrast, M2 TAMs release immunosuppressive cytokines, such as CCL2, CCL17, CCL18, CCL22, and CCL24 which drive a reduction of cytotoxic T cell activation and proliferation to promote tumor progression (Mantovani et al., 2013; Shapouri-Moghaddam et al., 2018). In addition, M2 TAMs have been reported to induce PD-L1 expression on tumor cells in non-small cell lung cancer (NSCLC) which prevents the activation of cytotoxic T cells (Sumitomo et al., 2019; Shima et al., 2020).

### Dendritic Cells (DCs)

DCs are antigen-presenting cells that make up a subtype of mononuclear phagocytes that regulate adaptive immune response; as such, DCs are necessary for T-cell-mediated cancer immunity. DCs are derived from hematopoietic bone marrow progenitor cells. These progenitor cells initially transform into DC precursors (pre-DC) and plasmacytoid dendritic cells (pDC) in the blood and spleen. The pre-DC can also differentiate into the mature form of DCs: mDC-1 and mDC-2. In recent studies, single cell RNA-seq allowed for the identification of novel surface markers to identify subsets of DC populations (Villani et al., 2017; See et al., 2017). The presence of CD33 can discriminate preDCs from pDCs; preDCs express CD123^+^CD33^+^ and the CD123^+^CD33^−^ cells typified pDCs. In addition, the mature form of DCs, mDC-1 showed CD123^+^CD33^+^CADM^+^CD1c^−^ and mDC-2 expressed CD123^+^CD33^+^CADM^−^CD1c^+^ (See et al., 2017).

Early findings suggest that DCs exhibit opposing effects to TAMs in cancer progression. Indeed, the presence of tumor-infiltrating DCs has been associated with prolonged overall survival in patients with head and neck tumors, lung, bladder, and gastric carcinoma (Lotze, 1997). The function of pDCs infiltration into solid tumors is controversial. Reports supporting both a negative (Oshi et al., 2020) or positive (Conrad et al., 2012) effect on tumor progression can be found in the literature. Tumor-infiltrating DCs (TIDCs) have been found in the TME in many different cancer types, such as breast, colorectal, lung, renal, head and neck, bladder, gastric, and ovarian (Karthaus et al., 2012). Not surprisingly, TIDCs are believed to be a double-edged sword with respect to tumor progression depending upon whether the tumor is early or late in development. Recent studies suggest that mature TIDCs in the early stages of tumors exhibited anti-tumor immunity. However, in advanced stages of tumor, more immature TIDCs were found to promote tumors malignant progression (Kocián et al., 2011; Krempski et al., 2011; Scarlett et al., 2012).

### Neutrophils

Neutrophils are the most abundant type of leukocytes in humans (50–75%) and are the first recruited to infected or injured tissues by cytokines/chemokines and pathogen signals (Coussens and Werb, 2002). Neutrophils target microbes and prevent their dissemination through phagocytosis, generation of reactive oxidants, and neutrophil extracellular traps (NETs). In recent years, the neutrophil-to-lymphocyte ratio has been reported to reflect cancer-related inflammatory responses and have prognostic value with respect to survival in patients with colorectal cancer (Walsh et al., 2005), non-small cell lung cancer (Sarraf et al., 2009), breast cancer (Ethier et al., 2017), prostate cancer (Kawahara et al., 2020), and hepatocellular carcinoma (Tada et al., 2020). Some evidence suggests a high NLR and high level of circulating neutrophils in patients with pancreatic cancer is associated with an increased number of tumor-associate neutrophils (TAN) within the TME (Takakura et al., 2016).

Like macrophages, tumor-associate neutrophils (TANs) can be divided into two subgroups: N1, which exist anti-tumor phenotype and N2, which exist pro-tumor phenotype following cytokine/chemokines stimulation (Fridlender et al., 2009; Fridlender and Albelda, 2012). N1 TANs release pro-inflammatory cytokines, including IL-12, CCL3, and CXCL9, which facilitate recruitment and activation of CD8^+^ T cells (Fridlender and Albelda, 2012; Coffelt et al., 2016). In contrast, N2 neutrophils promote tumor angiogenesis, metastasis by mediated matrix metalloproteinase (MMPs) (Dumitru et al., 2013; Coffelt et al., 2016), and neutrophils elastase (NE) released from the TANs (Brandau et al., 2013). It has been reported that N1 or N2 TANs can be derived from the other subtype during tumor progression (Mishalian et al., 2013). In the presence of TGF-β or GM-CSF microenvironment, neutrophils are polarized toward the N2 phenotype, whereas the blockade of TGF-β facilitates neutrophil development into an N1 phenotype (Fridlender et al., 2009; Casbon et al., 2015; Coffelt et al., 2016; Shaul et al., 2016). In contrast, type I interferons polarize neutrophils to an N1 phenotype while impaired type I interferon signaling results in polarization of neutrophils to an N2 phenotype (Pylaeva et al., 2016).

Tumor-associate neutrophils (TANs) have been shown to enhance tumor progression through NET formations (NETs) (Cedervall and Olsson, 2016). NETs occur when neutrophils extrude a meshwork of chromatin DNA fibers and release specific cytotoxic enzymes, such us myeloperoxidase (MPO), to trap and kill invading pathogens (Brinkmann et al., 2004). Findings suggest that NETs are highly associated with metastasis (Cools-Lartigue et al., 2013; Park et al., 2016; Tohme et al., 2016; Albrengues et al., 2018; Yang et al., 2020) and cancer-related thrombosis in different cancer types (Demers et al., 2012; Fuchs et al., 2012). *In vivo* studies suggest that depletion or inhibition of NET formation significantly reduces tumor metastases in lung cancer (Cools-Lartigue et al., 2013), ovarian cancer (Lee et al., 2019a), colorectal cancer (Tohme et al., 2016), pancreatic cancer (Miller-Ocuin et al., 2019), and breast cancer (Al-Haidari et al., 2019). In addition, a recent study has shown that NETs can activate CCDC25 on cancer cells and enhance cell motility (Yang et al., 2020). It had been showed that CXCR2 ligands, such as C-X-C motif chemokine (CXCL)1–3 and CXCL5–8 released from various cancer type drive neutrophil recruitment from circulation and chemotaxis toward the tumor (Sharma et al., 2013). In addition, CXCL5, a neutrophil activating chemokine is associated with poor prognosis in patients with HCC (Zhou et al., 2012). Our recent work demonstrated a similar finding when we found that CXCL5 released from pancreatic cancer cells mediated CD11b^+^Ly6G^+^ neutrophil infiltration into the TME. In addition, CXCL5 induced these neutrophils to form NETs that aided cancer cell invasion and metastasis (Deng et al., 2021). Moreover, additional studies have demonstrated that NETs can trap circulating tumor cells (CTCs) to facilitate metastasis (Cools-Lartigue et al., 2013; Najmeh et al., 2017). NETs can cover CTCs with activated platelets and facilitate metastatic events by creating a physical barrier that allows CTCs to escape CD8^+^ T cell- and natural killer cell-mediated cytotoxicity (Teijeira et al., 2020; Ren et al., 2021). These recent findings suggest that targeted disruption of the interaction between platelets, tumor cells and NETs holds therapeutic promise to prevent postoperative distant metastasis.

### Myeloid-Derived Suppressor Cells

MDSCs are derived from myeloid progenitor cells; under various pathologic conditions, such as infection, chronic inflammation, traumatic stress and cancer, myeloid progenitor cells fail to differentiate into their final mature form (Anani and Shurin, 2017). Within solid tumors, the accumulation of MDSCs within the TME correlates with the presence of tumor-mediated cytokines/chemokines, including IL-6, IL-1, GM-CSF, prostaglandins, and TGF-β, correlated with clinical tumor stage (Almand et al., 2001; Mirza et al., 2006; Ochoa et al., 2007; Diaz-Montero et al., 2009). MDSCs can be divided into two populations based on whether their origin is derived from granulocytic or monocytic myeloid cell lineages: granulocytic/polymorphonuclear MDSCs (PMN-MDSCs) and monocytic MDSCs (M-MDSCs).

It is difficult to discriminate PMN-MDSCs from neutrophils since they both share the same cell surface phenotypes (CD11b^+^Ly6G^+^Ly6C^−^ cells in mice and CD66b^+^CD14^−^CD11b^+^CD15^+^ cells in humans) (Bronte et al., 2016). PMN-MDSCs are often labelled “low density neutrophils” (Bronte et al., 2016; Mishalian et al., 2017); PMN-MDSCs possess features typical for N2 TAN and are known to exert a locally immunosuppressive effect within the TME associated with cancer progression (Fridlender et al., 2009; Fridlender et al., 2012). Phenotypically, M-MDSC are HLA-DR^−^CD11b^+^CD33^+^CD14^+^CD15^−^ in humans and Gr-1^+^CD11b^+^Ly6C^+^Ly6G^−^ in mice (Bronte et al., 2016). Although the two populations of MDSCs express different surface markers, all MDSCs are believed to inhibit immune responses and promote tumor progression (Dietlin et al., 2007; Movahedi et al., 2008; Youn and Gabrilovich, 2010).

MDSCs facilitation of cancer metastasis events occur through direct and indirect means. PMN-MDSCs promote metastasis through the production of producing proteases, such as matrix metalloproteinases (MMPs), that degrade ECM to facilitate cancer cell extravasation and promote engraftment of CTCs at metastatic sites (Spiegel et al., 2016). M-MDSCs inhibit IL-12 production from macrophages while stimulating IL-10 which leads to a shift from M1 to M2 TAMs to further decrease immune response and facilitate tumor progression (Trinchieri, 2003).

MDSCs negatively influence the host immune function within the TME through numerous mechanisms including the production of oxidative stress, inhibiting the effective migration of antitumor effector cells, and increasing regulatory T (Treg) cells (Gabrilovich et al., 2012). MDSCs-mediated ROS and RNS production directly inhibit T cells by nitrating T cell receptors (TCRs) and reducing their responsiveness to cognate antigen-MHC complexes (Nagaraj et al., 2007). Furthermore, RNS-mediated nitration or nitrosylation chemokines block the migration and infiltration of T cells (Molon et al., 2011). A recent study suggests that MDSCs-mediated TGF-β and IL-10 play an important role in developmental of CD4^+^ CD25^+^ Treg cells, which can significantly suppress antigen-specific immune responses. (Huang et al., 2006).

## Cancer and Host Factors Contributing to Inflammation

Virchow first suggested that the origin of cancer was at sites of chronic inflammation in 1863 (Balkwill and Mantovani, 2001). The Cancer Genome Atlas (TCGA) consortium comprised 33 different cancer types and identified some immune signatures that modules dominating specific tumor types (Thorsson et al., 2018). Not surprisingly, tumors within lower T lymphocytes and NK cells microenvironment, such as uveal melanoma and brain tumors, contributes to their limited response to immune checkpoint blockade (Heppt et al., 2017). Conversely, lung carcinoma and skin melanoma showed the highest immune cell fraction include malignancies most responsive to immunotherapy (Thorsson et al., 2018). However, some type of tumors, such as pancreatic cancer, exist strong lymphocytes infiltration has been shown to be restricted the activity of T cells by myeloid-inflamed stroma, which contributed to the poor responses to immunotherapy (Beatty et al., 2015; Tsujikawa et al., 2017).

**FIGURE 2:**
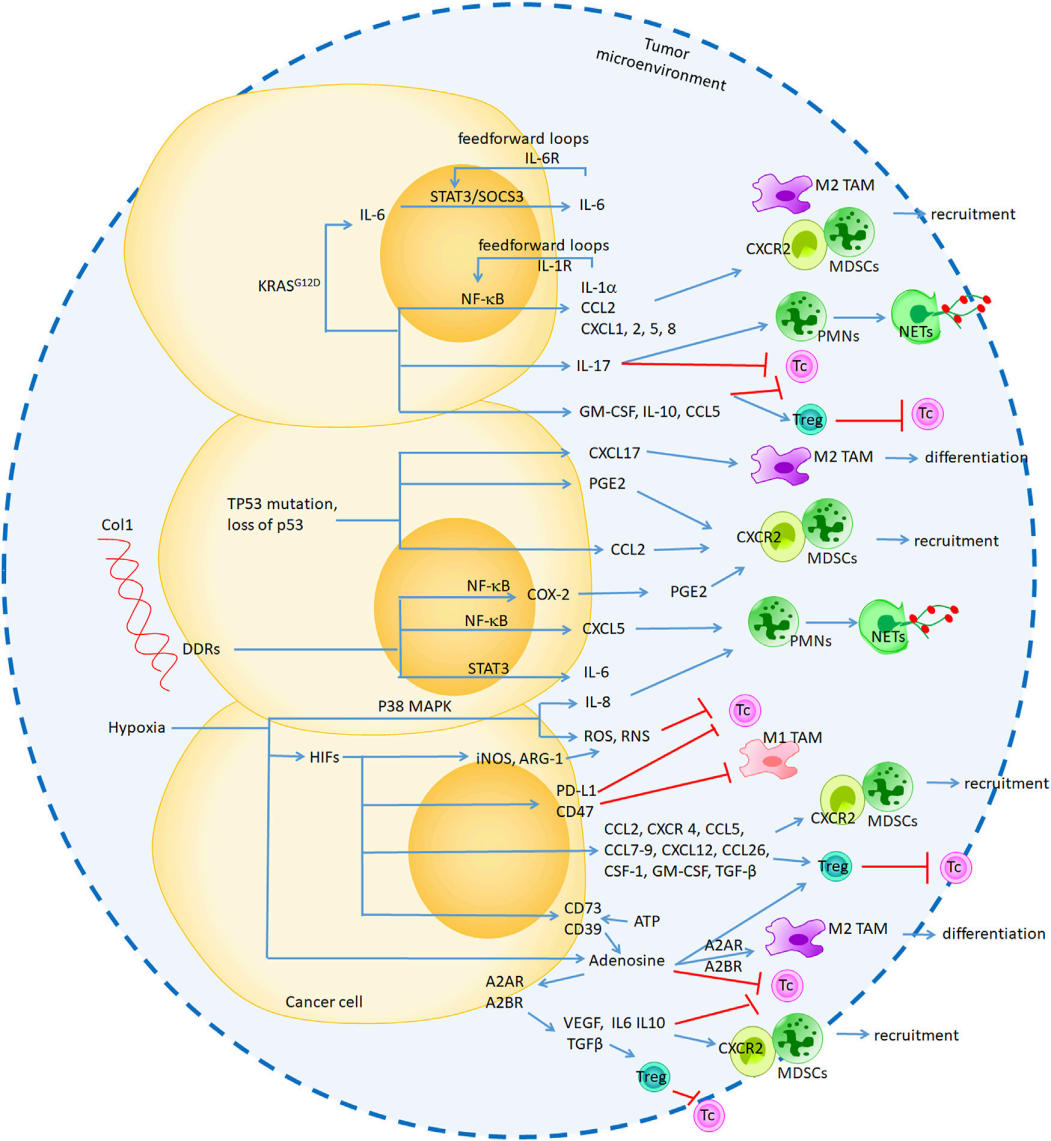
The genetic and environmental conditions that trigger inflammatory signaling cascades, such cyclooxygenase-2 (COX-2), NF-κB and STAT3, to induce secretome production and influence tumor-associated myeloid cells within the TME. Genetic KRASG12D and TP53 mutation regulated CXCR2, CCR5 ligands secretion through NF-κB and STAT3 pathway to promote MDSCs accumulation and immunosuppression. Environmental collagen-DDR1 regulated CXCL5 through NF-κB pathway to induced NETs formation. Hypoxia mediated HIF regulated CXCR2, CXCR4 and CCR5 ligands production, which induced MDSCs recruitment and Tregs generation. In additiob, HIF-regulated ROS and RNS as well as “don’t eat me “signals, which directly inhibited pro-inflammatory response. Hypoxia-mediated adenosine not only directly regulated M2 TAM and Tc cells by binding its receptor A2A/A2B, but also regulated cancer cells-mediated VEGF, LI-6, IL-10 and TGF-β secretion to promote DMSCs recruitment.

Experimental studies have demonstrated that chronic stress, including cytokines, chemokines and secretomes regulated the tumor-associated myeloid microenvironment. Here, we summarized the genetic and environmental conditions that trigger inflammatory signaling cascades, such cyclooxygenase-2 (COX-2), NF-κB, and STAT3, and influence tumor-associated myeloid cells within the TME (Figure 2).

**FIGURE 3:**
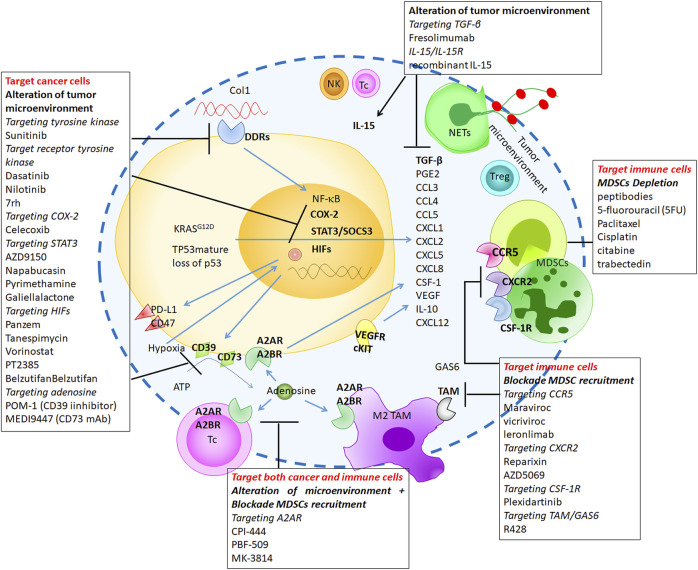
The promising strategies targeting immunosuppressive-myeloid cells through cell depletion, blockade of known immunosuppressive signaling or alteration of TME.

### TP53

Point mutations within the *p53* gene have been detected in many cancer types including 30% of GBM, 67.4% of pancreatic cancer, and up to 90% of ovarian serous cystadenocarcinoma and pulmonary squamous cell carcinomas (Cancer Genome Atlas Resea, 2011; Cancer Genome Atlas Resea, 2012; Kanda et al., 2013). Studies have identified that stromal-specific loss of p53 acts through CXCL17 to effect M2 TAMs differentiation, MDSCs infiltration, and CD11b^+^Gr1^+^ MDSCs recruitment that conspire to enhance tumor growth (Ryan et al., 2000; Guo et al., 2013; Bezzi et al., 2018). In addition, tumor-specific loss of p53 in lung and pancreatic cancer promotes immune tolerance through regulation of immunosuppressive myeloid cell recruitment (Blagih et al., 2020). The specific molecular mechanism for this remains unclear and may depend upon the whether the gene mutation leads to increased function or loss of the protein product. Mutant *p53* activates the NF-κB pathway leading to immune cell apoptosis through tumor-necrosis factor-α and activation of pp90^rsk^ (Ryan et al., 2000). These gain-of-function *p53* mutations and NF-κB pathway activation also drives secretion of the chemokine CCL2 that leads to tumor infiltration by immunosuppressive myeloid cells (Ham et al., 2019). TP53 was also been showed to mediate COX-2 activity suggesting that loss of *p53* or *p53* mutation triggers an immunosuppressive effect through the NF-κB-CCL2 and COX-2-PGE2 pathways (Gallo et al., 2003).

### Kirsten Rat Sarcoma Viral Oncogene Homolog

Oncogenic activating point mutations in codon 12, 13, and 61 of the *Kras* gene are present in about 63% non-small cell lung cancers (Slebos et al., 1990), about 50% colorectal cancers (Porru et al., 2018) and 70–95% pancreatic cancers (Buscail et al., 2020). *Kras* mutations have been tightly linked to tumor-mediated inflammation and tumorigenesis through several induced inflammatory cytokines, chemokines (Golay and Barbie, 2014; Kitajima et al., 2016). Both the JAK-STAT3 and NF-κB pathways are involved in Kras induced cytokine/chemokine production. IL-6, which induces STAT3/SOCS3 activity in a paracrine or autocrine fashion, is upregulated by mutant Kras activity, and genetic deletion of IL-6 or STAT3 can prevent mutant Kras-mediated pancreatic tumorigenesis (Lesina et al., 2011). Similarly, higher elevated levels of CXCL8 have been observed in the serum and tumor tissues in patients with Kras mutant-pancreatic and lung cancer (Wigmore et al., 2002; Tas et al., 2006; Frick et al., 2008). In lung cancer, mutant Kras is known to drive IL-8 release from cancer cells via activation of the AP-1 and NF-κB transcription pathways; IL-8 in the TME drives M2 macrophage polarization and neutrophil recruitment to promote progression (Wislez et al., 2006). In pancreatic cancer, mutant Kras drives release of CXCL1, 2 and 5, which are CXCR2 ligands necessary for angiogenesis, myeloid cell infiltration and tumor progression (O'Hayer et al., 2009; Li et al., 2011). Conversely, global deletion of CXCR2 in Kras^G12D/+^p53^R172H/+^ transgenic (KPC) mice significantly increased T cell infiltration and prevented metastasis in animal models of pancreatic cancer (Steele et al., 2016). Constitutive Kras and NF-κB activation are signature alterations in PDAC (Bailey et al., 2016), and KrasG12D-activated IL-1α/NF-κB/IL-1α and p62 feedforward loops are necessary for the induction and maintenance of NF-κB activity (Ling et al., 2012). In other studies, Kras^G12D^-dependent production of GM-CSF, IL-10, and CCL5, which recruit MDSCs and convert CD4+ T cells to Treg cells, play an important role in cancer progression (Karnoub et al., 2007; Pylayeva-Gupta et al., 2012; Li et al., 2018). Recent studies in pancreatic cancer identified activation of IL-17/IL-17R pathway driven by Kras^G12D^ caused a subsequent decrease in cytotoxic T cell recruitment into the TME and an increase in neutrophil recruitment and NETs (McAllister and Leach, 2014; Zhang et al., 2020a). Together, these studies support an important role for Kras mutant in establishing an immunosuppressive microenvironment.

### MAPK Pathway

Mitogen-activated protein (MAP) kinases, including ERKs, JNKs, and p38s, are regulated by a phosphorelay cascade have been shown to contribute with chronic inflammation and inflammation-associated cancer progression (Huang et al., 2010).

P38 MAPK has been activated expression after cellular stress or cytokine signaling (Bradham and McClay, 2006). Hypoxia-induced activation of p38 MAPK signaling contributes to expression of IL-8 in human ovarian carcinoma cells (Xu et al., 2004). In addition, p38 MAPK is activated by ROS and RNS produced by hypoxia in various human disease states (Chandel et al., 2000; Powell et al., 2004; Sumbayev and Yasinska, 2005). Lastly, inhibition of the p38 MAPK pathway is associated with reduced release of TNFα and IL-1β suggesting that p38 MAPK pathway regulats inflammatory response through cytokine production (Zheng et al., 2016).

Recent studies revealed that ERK also promotes inflammation-associated cancer development, mainly through regulating the expression of inflammatory cytokines. immunosuppressive soluble factors IL-10, VEGF, IL-6 and IL-8 were demonstrated stimulated by activation RAS-Raf-MEK-ERK signaling. Constitutive activation of the ERK cascade is required for CXCL-8 transcription and contributes to maximal IL-8 gene expression (Sparmann and Bar-Sagi, 2004; Sumimoto et al., 2006). In addition, activation of MAPK signal, along with the STAT3 signal, is essential for immune evasion in human melanomas by increasing IL-6, IL-10, and VEGF production (Sumimoto et al., 2006).

JNK-MAPK signaling has been linked to the expression of metalloproteinases and inflammatory cytokines that control cancer progression (Karin and Gallagher, 2005). Recent work suggests that JNK signaling might link cell-specific ER stress to the induction of organ-specific inflammation and subsequent cancer development (Kaser et al., 2008).

### Collagen

Collagen is the principle structural protein in the ECM with fibrillar collagens as the most abundant types in healthy tissues. In pathogenic conditions, such as cancer, collagen accumulates in the affected tissue and is a major component of fibrosis associated with cancer. In solid tumors, collagen-driven fibrosis appears both restrain and promote tumor progression depending upon the stage of cancer development. Collagen forms fibrous networks around the cancer cells within the TME and these can act as “stromal highways” which support cancer cell migration and proliferation (Armstrong et al., 2004; Hanahan and Weinberg, 2011; Inoue et al., 2016; Chen et al., 2021). In addition, fibrillar collagen fiber density within the TME correlates positively with tumor stage (Wei et al., 2017; Lee et al., 2019b). Lastly, data from many high-quality mechanistic studies suggest that Collagen promote metastasis in breast carcinoma (Provenzano et al., 2008), bladder tumors (Lee et al., 2019b), and pancreatic cancer (Shintani et al., 2006).

In previous studies suggest that Collagen receptors on the surface of cancer cells, such as integrins, Discoidin Domain Receptor (DDRs), and the mannose receptor, play an important role in Collagen-mediated tumor progression. DDR1 is a receptor tyrosine kinase is activated by Collagen-induced COX-2 and promotes chemoresistance through the NF-κB pathway (Das et al., 2006). Our recent study also found that Collagen I triggers CXCL5 production through a DDR1-NF-κB pathway; CXCL5 then acts to recruit neutrophils into the TME where they form NETs and induce pancreatic metastasis (Deng et al., 2021). In addition to NF-κB, the STAT3 pathways are also involved in DDR1 downstream signaling and cytokines/chemokines production (Seo et al., 2008; Gao et al., 2016; Roca et al., 2018). The loss of Collagen is also associated with cancer progression. A recent study found that deletion of Collagen I in myofibroblasts was associated with increased number of CD206^+^ F4/80^+^ Arg1^+^ MDSCs and contributed to the immunosuppressive microenvironment in PDAC (Chen et al., 2021). Another two studies suggest that CD206 (mannose receptor)-expressing M2 TAMs bind to collagen leading to collagen degradation through cellular uptake in a CCR2-dependently pathway (Madsen et al., 2013; Madsen et al., 2017). Taken together, these studies clearly link cancer progression with the status and interactions of collagen with the cancer cell compartment within the TME. Current concepts suggest that in early tumor development, collagen secreted from fibroblasts surround the cancer cells and restrain against tumor growth. However, as the tumor mass grows, collagen-cancer cell physical interaction drives signaling that drives the TME toward immunosuppression where M2 TAMs then degrade collagen. Indeed, our pervious analysis of pancreatic cancer tumor stroma identified worse cancer-related outcomes in patients with high cancer cell content and decreased collagen in the TME (Koay et al., 2018).

### Growth Arrest-specific 6/Protein S

Gas6 is a 75 kDa vitamin K-dependent protein that was originally described as secreted from fibroblasts. High levels of Gas6 have been detected within the TME of various cancer types, including lung, gastric, pancreatic cancer and hepatocellular carcinoma (Wu et al., 2017). Gas6 is a ligand for homologous type 1 receptor tyrosine kinase receptors, Tyro3, Axl, and Mer receptors (collectively referred to as TAM). Current understanding is that tumor-associated macrophages are the primary source of Gas6 within the TME (Wu et al., 2018). Gas6/TAM binding then triggers receptor activation and numerous downstream signaling pathways on both cancer and host cells. Importantly, Gas6/TAM activation is implicated in M2 TAMs polarization through the Axl/PI3K/Akt/NF-κB pathway (Chiu et al., 2015). In addition, crosstalk occurs between activated TAM and the interleukin (IL)-15 receptor to drive additional pathways including NF-κB pathway, and STAT signaling to regulate transcription of important cytokines/chemokines (Hafizi and Dahlback, 2006; Linger et al., 2008). A driving force in this cascade is tumor cell-derived IL-10 and M-CSF, which attracts and modifies circulating lymphocytes in the TME to secrete Gas6 into the TME (Loges et al., 2010).

### Hypoxia

Hypoxia is a relevant physioligical stress associate with many human disease, such as COPD and Cancer (Semenza, 2000). The relationship between hypoxia and inflammation occurs through multiple molecular mechanisms that link hypoxia-induced inflammation in cancer progerssion (Lukashev et al., 2007; Balamurugan, 2016; Ke et al., 2019; Allard et al., 2020). Hypoxia-inducible factors (HIFs) are oxygen-sensitive transcription factors that comprise a critical component in the cellular response to an hypoxic environment with HIF-1α being the most associated with malignancy (Semenza and Wang, 1992). Hypoxia-induced activation of the NF-κB pathway was first reported in 1994 (Koong et al., 1994) and numerous studes have since linked NF-κB and HIFs. These studies demonstrate that NF-κB regulates HIF-1α through regulation of gene transcription and protein stabilization (van Uden et al., 2008; van Uden et al., 2011). When the TME is under hypoxic conditions, HIF-1α acts to increase inducible nitric oxide synthase (iNOS) and arginase (ARG-1), which inhibit cytotoxic T cell migration and function (Corzo et al., 2010; Noman et al., 2015). In addition, HIF-1α triggers expression of “don’t eat me” proteins, including CD47 and PD-L1, on the surface of cancer cells allowing them to escape phagocytosis and adaptive immunity (Barsoum et al., 2014; Zhang et al., 2015). HIF also promotes an immunosuppressive microenvironment through the production of various cytokines/chemokines, such as CCL2, CXCR 4, CCL5, CCL7-9, CXCL12, and CCL26 as well as CSF-1, GM-CSF, and TGF-β, that recruit PMN-MDSCs and macrophages to the TME that inhibit NK cell activity (Borsig et al., 2014; Schito and Semenza, 2016).

### Adenosine

Adenosine is another molecule that is present in the tumor microenvironment modulates the anti-cancer immune response in hypoxic conditions. Adenosine is generated in tumors through the coordinated activity of the ectonucleotidases CD39 (ENTPD) and CD73 (NT5E) that together convert extracellular adenosine triphosphate (ATP) to adenosine (Ohta and Sitkovsky, 2001; Antonioli et al., 2013). Extracellular adenosine inhibits the proinflammatory effects through G-protein-coupled adenosine receptors, A1, A2a, A2b, and A3 which suppresses antitumor immunity (Sheth et al., 2014). It had been reported that HIF1α-driven CD73 and CD39 transcriptional activation in epithelia and endothelia provides an important link for hypoxia-mediated adenosine production (Synnestvedt et al., 2002). The role of adenosine in cancer progression contributed to the induction immunosuppressive phenotypes, including regulation of cytotoxic T cell migration and function, Treg cell generation, M2 macrophage differentiation, and the production of tolerogenic DCs, PMN-MDSCs via the A2A or A2B receptor pathway (Novitskiy et al., 2008; Young et al., 2014). In addition, activation of A2A receptor signaling by adenosine enhances the release of important cytokines, such as VEGF, IL-6, IL-10, and TGFβ, which suppress the inflammatory response and enhance tumor survival (Young et al., 2014; Ansari et al., 2017).

## Therapeutic Strategies Targeting Myeloid Cells

The recent promising advances in the field of immunotherapy in oncology have focused on cytotoxic T cell killing of cancer cells. While immunotherapy holds promise as a therapy to cure patients with cancer, the results in clinical studies have been variable demonstrating our incomplete understanding of the complex mechanisms in play. New information from the investigation of myeloid cells and inflammation in cancer suggest cells from this lineage can promote cancer progression and resistance to control T cell activity. Myeloid cells within the TME are also associated with resistance against treatments, reducing the efficacy of immunotherapies, and ultimately in patient outcomes (Nakamura and Smyth, 2020). Here, we will summarize promising strategies targeting immunosuppressive-myeloid cells through cell depletion, blockade of known immunosuppressive signaling or alteration of TME (Figure 3).

### Depletion of Immunosuppressive-Myeloid Cells

Low dose chemotherapy such as 5-fluorouracil (5FU), paclitaxel, cisplatin, citabine has been shown to be effective in depletion of neutrophils and MDSCs (Vincent et al., 2010; Sevko et al., 2013). The chemotherapeutic agents can also be cytotoxic for monocytes and macrophages such as trabectedin, a DNA-damaging agent (Mantovani and Allavena, 2015). Conversely, it has been demonstrated that TAMs generally accumulate in tumors after chemotherapy and contribute to protect tumor cells survival by inhibiting cell death signaling pathways (De Palma and Lewis, 2011; Hughes et al., 2015). In Lewis lung carcinoma model (LLC1s) and mouse models of breast cancer metastasis (MMTV-PyMT), treatment with cyclophosphamide, paclitaxel and doxorubicin significantly increase CD206^+^ TAMs accumulation arround CXCL12-rich tumor cells, suggested that tumor-mediated CXCL12 recruits CD206^+^ TAMs resulting in chemoresistance (Hughes et al., 2015). S100 family proteins are expressed on the surface of MDSCs and may represent a new target for cancer immunotherapy. S100-specific peptide-Fc fusion proteins (termed peptibodies) were shown to effectively deplete MDSCs in various cancer types in tumor-bearing mice without effecting other immune cells (Hughes et al., 2015).

### Blockade the Immunosuppressive Cells Recruitment and Infiltration

Inhibition of immunosuppressive myeloid cell recruitment is a therapeutic strategy in which surface chemokine receptors on the surface of myeloid cells are blocked to prevent ligand binding and subsequent migration and activation. **CCR5**, expressed on MDSC, TAMs, and Treg cells, plays a central role in the chemotaxis of MDSC into the tumor microenvironment via the ligands CCL3, CCL4, and CCL5 (Weber et al., 2018). Importantly, MDSCs that express CCR5 have more potent immunosuppressive mechanisms compared to the ones that do not (Blattner et al., 2018). In addition, blockade of CCR5 significantly inhibits MDSCs recruitment and prevents cancer metastasis (Velasco-Velázquez et al., 2012). Several CCR5 antagonists developed for HIV treatment are being retasked for cancer and cancer-related diseases (Fätkenheuer et al., 2008). Maraviroc, vicriviroc, and leronlimab prevent breast cancer metastasis by increasing the chemosensitivity (Velasco-Velázquez et al., 2012; Jiao et al., 2018). Three clinical trials, which target CCR5 in combination with FDA approved drugs showed the beneficial effect in cancer patients. A phase I study of pembrolizumab with maraviroc is ongoing in patients with colorectal cancer; A phase II study of vicriviroc in combination with pembrolizumab is ongoing in patients with advanced metastatic MSS-colorectal cancer; and a phase Ib/II study of carboplatin and leronlimab is designed for patients with CCR5^+^ metastatic triple negative breast cancer (Jiao et al., 2019).

During cancer progression, cancer cell-derived CCL2, CCL5, CXCL8, and CXCL5 are released and recruit immunosuppressive-myeloid cells into the TME, including MDSCs, TAMs, and TANs through **CXCR2** activation (Toh et al., 2011; Katoh et al., 2013; Xiao et al., 2015; Zhang et al., 2020b). Earlier studies demonstrated that genetic knockout or small-molecule inhibition of CXCR2 blocks recruitment of MDSCs into tumors and enhances the efficacy of cyotoxic therapy and anti-PD1 immunotherapy to improve the outcomes of patients with sarcoma, head and neck, pancreatic, and liver cancer (Highfill et al., 2014; Steele et al., 2016; Greene et al., 2020). CXCR2 inhibitors in clinical trials include: Reparixin in a Phase 2 clinical trials for patients with triple-negative breast cancer (NCT02370238); and AZD5069 in Phase 1b/2 trial for patients with advanced solid tumors (NCT02499328) (Sharma et al., 2013; Law et al., 2020).

Signaling via **CSF-1R**, a tyrosine kinase receptor drives differentiation and expansion of MDSCs and TAMs and promotes their migration into tumors (Holmgaard et al., 2016). In various cancers, such as pancreatic and breast, cancer-associated MDSCs express high levels of surface CSF-1R which is responsible for immunosuppressive effects through inhibition of T cell activity (Sluijter et al., 2014; Zhu et al., 2014). Treatments targeting the CSF-1 receptor inhibit TAMs and MDSCs recruitment that improves T cell activity and prevents tumor progression. (Mok et al., 2014; Zhu et al., 2014). In addition, combination of CSF-1R and CXCR2 inhibitors resulted in strong anti-tumor effect (Kumar et al., 2017). Pharmacodynamics studies have shown that plexidartinib, a CSF-1R inhibitor, which is in phase 2 against glioblastoma also reduced circulating monocytes (CD14^+^/CD16^+^) and increased serum CSF-1 (Butowski et al., 2016).

**TAM receptors**, expressed on macrophages, promote M2 polarization and efferocytosis which are tumor-promoting processes (Lu et al., 1999). R428, an Axl-selective inhibitor, has been shown to block breast cancer cell invasion and GM-CSF production (Holland et al., 2010). R428 is currently in clinical trials for patients with breast cancer, non-small cell lung cancer (NCT02922777, NCT02424617), acute myeloid leukemia (NCT02488408), and metastatic melanoma (NCT02872259) (Janning et al., 2015).

### Alteration of the Tumor Microenvironment

**TGF-β** is secreted from tumor cells and drives immunosuppression within the TME through numerous actions: PMN-MDSCs recruitment, M2 polarization of TAMs and Treg differentiation (Grutter et al., 2008). Anti-tumorigenic effects against melanoma and renal cell cancer have been observed in ongoing trials testing fresolimumab, a monoclonal antibody against TGF-β (Grutter et al., 2008; Colak and Ten Dijke, 2017).

Systemic delivery of **IL-15**, a cytokine similar to IL-2, triggers proliferation of NK cells and enhances anti-tumor immunity of CD8 T cells in several preclinical animal model studies (Steel et al., 2012; Yu et al., 2012). A phase I trial of recombinant IL-15 in adults with refractory metastatic malignant melanoma and metastatic cancer also demonstrated that IL-15 increased the activity of NK and CD8 T cells (Conlon et al., 2015). Romee et al., found that preactivation with IL-15 and IL-12 enhanced memory-like NK cells resulting in an increased IFNγ production and cancer cell cytotoxicity in patients with primary acute myeloid leukemia (Romee et al., 2016). Additionally, recent phase I clinical studies found that ALT-803, an IL-15 superagonist, increased the number of circulating NK cells resulting in enhanced pro-inflammation and tumor growth arrest in patients with advanced sold tumors (Fantini et al., 2019).

Sunitinib is an FDA-approved tyrosine kinase inhibitor that blocks VEGFR, PDGFR and c-KIT signaling, all of which have been implicated in cancer growth, pathologic angiogenesis, and metastatic progression. In recent patient studies, sunitinib has been demonstrated to deplete MDSCs accumulation through a reduction in STAT3 signaling activity within renal cell carcinoma and breast cancer (Ko et al., 2009; Kodera et al., 2011).

Nilotinib is another tyrosine kinase receptor inhibitor that blocks multiple pathways and is FDA-approved for use in patients with chronic myelogenous leukemia. Recently it has been shown to inhibit phosphorylation of **DDR1** inhibitor with preclinical effectiveness against colorectal cancer (Karaman et al., 2008; Jeitany et al., 2018). In recent work, we also demonstrated a similar effect in preclinical studies in pancreatic cancer using another DDR1 inhibitor, 7rh (Aguilera et al., 2017). We found that treatment with 7rh interrupts collagen I-mediated CXCL5 production from cancer cells, which result in NETs formation and reduced TAN recruitment (Deng et al., 2021).

**Prostaglandin E2** (PGE2) is an important, well-studied inflammatory mediator that is also associated with MDSCs recruitment and an immunosuppressive TME (Sinha et al., 2007; Eruslanov et al., 2010). Celecoxib, an FDA-approved non-steroidal anti-inflammatory drug (NSAID), is a COX-2 selective inhibitor that reduces MDSCs recruitment, inhibits MDSC-mediated immunosuppressive effect and can improve immunotherapy in mesothelioma patients (Eruslanov et al., 2010; Veltman et al., 2010; Zelenay et al., 2015).

Targeting the transcription factor **STAT3** is another clinical strategy. AZD9150, a STAT3 antisense oligonucleotide reduces PMN-MDSCs within the peripheral blood mononuclear cells in patients with diffuse large B-cell lymphoma (Reilley et al., 2018). Napabucasin (BBI608) is an FDA approved orally administered small molecule that blocks stem cell activity in cancer cells by targeting the STAT3 pathway. In clinical trials, napabucasin prevented tumor metastasis when used alone or in combination (Hubbard and Grothey, 2017). Pyrimethamine (PYR) is an FDA-approved anti-microbial drug that is also an inhibitor of STAT3 function at concentrations that can be achieved safely in humans. PYR shows therapeutic activity in mouse models with breast cancer where it reduced tumor-associated inflammation increased tumor-infiltrating CD8+ T cells and activity (Khan et al., 2018). Galiellalactone is a direct inhibitor of STAT3 that inhibits DNA binding and IL-6 dependent signaling. In prostate cancer preclinical studies, galiellactone inhibits cancer cell-associated MDSCs and reverses the immunosuppressive mechanisms caused by the interplay between prostate cancer cells and MDSCs. (Hellsten et al., 2019).

Many drugs that specific target **HIF** are in clinical trial or are already approved for the treatment of cancer. For example, 2-methoxyestradiol (Panzem) inhibits HIF-1a protein synthesis and transcriptional activity has been used in combination with bevacizumab (VEGF mAb) in a Phase II clinical trial for metastatic carcinoid neuroendocrine tumor, result in reduced the tumor size in 68% of cancer patients (Kulke et al., 2011). 17-AAG (tanespimycin) is derived from the antibiotic geldanamycin and is a potent inhibitor of heat shock protein 90 (HSP90), which increases degradation of the HIF-1a. Human trials demonstrate a reduction in tumor size in 39% of patients with lymphoma (Oki et al., 2012), but not in patients with RCC (Ronnen et al., 2006) and metastatic prostate cancer (Heath et al., 2008). Vorinostat is a histone deacetylase inhibitor that suppresses the HIF pathway through enhanced degradation of the HIF-1α and was recently approved by the FDA for treatment of patients with cutaneous T cell lymphoma (Mann et al., 2007). PT2385 is a HIF-2α antagonist that inhibits dimerization of the HIF-2α and binding to DNA. Phase II clinical trials (NCT03108066 and NCT03216499) have shown clinical benefit in 66% of patients with metastatic renal cell carcinoma (Courtney et al., 2018). Belzutifan (MK-6482) is another HIF-2α inhibitor with a mechanism of action similar to PT2385. A phase II trial demonstrated that MK-6482 is well tolerated and has promising single-agent activity in heavily pretreated patients with RCC (Hasanov and Jonasch, 2021).

Treatment strategies targeting **adenosine** have attempted to block both the production of adenosine and adenosine binding to its receptors. There are currently four agents targeting the A2a receptor for cancer immunotherapy in Phase 1 trials, including CPI-444 (Corvus), PBF-509 (Novartis/Pablobiofarma), MK-3814 (Merck), AZD4635 (AstraZeneca/Heptares) (Leone and Emens, 2018). Treatment with CPI-444 alone or in combination with anti-PD-1 therapy with atezolizumab promoted cytotoxic T cell activity in RCC and NSCLC tumors (Willingham et al., 2018). NIR178 (PBF-509) is an oral A2AR antagonist that selectively binds and inhibits A2AR which reactivates T cell-mediated antitumor immune response. NIR178 is currently being evaluated in phase I/II studies in previously treated pts with advanced NSCLC (NCT02403193). CD 39 and CD73 are enzymes on the membrane surface of endothelial and immune cells that degrade ATP to immunosuppressive adenosine. Blockade of CD39 enzymatic activity may lead to an increase in extracellular ATP levels, enabling coactivation of inflammasomes which promotes tumor immunity. It has been demonstrated that CD39 inhibitors reestablished the proimmunogenic activity of anthracyclines in mice (Michaud et al., 2011). Sodium polyoxotungstate (POM-1) inhibits CD39 and restrains tumor growth and metastasis by increasing anti-tumor immunity (Sun et al., 2010). In addition, antibody-directed immunotherapies targeting CD73, MEDI9447 (Medimmune) demonstrated similar antitumor properties by increasing CD8+ T cell activity (Hay et al., 2016). In addition, combinations of MEDI9447 with other immune modulators: durvalumab (anti-PD-L1), tremelilumab (anti-CTLA-4), and MEDI0562 (anti-OX40) have been effective in a phase II trial of patients with ovarian cancer (Leone and Emens, 2018).

## Discussion

The immune system is a highly flexible system that is necessary to protect the organism from environmental injury and pathogens; however, cancer immunotherapy has to date been focused on a narrow aspect of this spectrum. The variable success and failure of this approach is not surprising given the strong immunosuppressive environment found in the microenvironment of many solid tumors. Immunosuppression is driven by molecular genetics of the cancer cells coupled with an inappropriate inflammatory and immune response. In this review, we discussed how myeloid cells contribute to this immunosuppressive effect and explored current strategies in development that could target this aspect of the immune response and improve cancer outcomes.
